# Identifying drug-pathway association pairs based on L1L2,1-integrative penalized matrix decomposition

**DOI:** 10.18632/oncotarget.18254

**Published:** 2017-05-29

**Authors:** Dong-Qin Wang, Ying-Lian Gao, Jin-Xing Liu, Chun-Hou Zheng, Xiang-Zhen Kong

**Affiliations:** ^1^ School of Information Science and Engineering, Qufu Normal University, Rizhao, China; ^2^ Library of Qufu Normal University, Qufu Normal University, Rizhao, China

**Keywords:** L_2,1_-norm, L_1_-norm, integrative penalized matrix decomposition, paired drug-pathway associations, drug discovery

## Abstract

The traditional methods of drug discovery follow the “one drug-one target” approach, which ignores the cellular and physiological environment of the action mechanism of drugs. However, pathway-based drug discovery methods can overcome this limitation. This kind of method, such as the Integrative Penalized Matrix Decomposition (iPaD) method, identifies the drug-pathway associations by taking the lasso-type penalty on the regularization term. Moreover, instead of imposing the L_1_-norm regularization, the L_2,1_-Integrative Penalized Matrix Decomposition (L_2,1_-iPaD) method imposes the L_2,1_-norm penalty on the regularization term. In this paper, based on the iPaD and L_2,1_-iPaD methods, we propose a novel method named L_1_L_2,1_-iPaD (L_1_L_2,1_-Integrative Penalized Matrix Decomposition), which takes the sum of the L_1_-norm and L_2,1_-norm penalties on the regularization term. Besides, we perform permutation test to assess the significance of the identified drug-pathway association pairs and compute the P-values. Compared with the existing methods, our method can identify more drug-pathway association pairs which have been validated in the CancerResource database. In order to identify drug-pathway associations which are not validated in the CancerResource database, we retrieve published papers to prove these associations. The results on two real datasets prove that our method can achieve better enrichment for identified association pairs than the iPaD and L_2,1_-iPaD methods.

## INTRODUCTION

With the rapid development of data generated from genetic analyses and functional genomics, identifying drug targets has become more and more feasible [1]. The modern drug discovery has found thousands of drug targets and the clinical testing agents. These agents can produce curative effects via regulating different targets in various biological and related diseases of pathways. Traditional drug discovery methods follow the “one drug-one target” line. But many complex diseases are related to dysfunction of multiple pathways rather than individual genes [2]. These methods ignore the relationship among genes and the systemic nature of human diseases. In general, pathways are defined as the interaction of multiple genes, that is, pathways can be regarded as the target of drugs [3, 4]. Abnormal biological pathways can provide views for the aberrant imbalance underlying diseases and find targets for complex diseases intervention [5].

Compared with the “one drug-one target” methods, the systematic biology approach takes the drug effects into the global physiological environment account [6]. These existing computational methods for identifying drug targets include the Gene Set Enrichment Analysis (GSEA) method [7], the FacPad method [2], the iFad method [5] and the iPaD method [8], etc. The GSEA method has several disadvantages. Firstly, for every paired drug-pathway association, calculation must be done once at every turn. Secondly, the genes which belong to the same pathway will have the common weights, therefore, when a subblock of genes serve as the critical interaction groups for a specific drug. In summary, the GSEA method is time-consuming and inaccurate. The FacPad method has been proposed to identify drug-pathway association pairs, and it develops a sparse Bayesian factor analysis model to deal with treatment response data that are derived from microarray platforms [2]. And the iFad method is also a sparse Bayesian factor model, which is proposed to infer the drug-pathway association using gene expression and drug sensitivity data. In literature [5], the authors apply this method on the NCI-60 data set. The gene expression and drug sensitivity data are downloaded from the CellMiner database [9] (http://discover.nci.nih.gov/cellminer). The iFad method is effective in identifying paired drug-pathway associations. Since this method uses the Markov Chain Monte Carlo (MCMC) [10] to perform statistical inferences, it is computationally expensive. Also it has many turning parameters which should be specified by users. In order to improve the algorithm speed and performance, a method named iPaD (integrative Penalized Matrix Decomposition) is proposed by Li et al.[8] to identify paired drug-pathway associations. The iPaD method applies the integrative penalized matrix decomposition method to analyze the gene expression data and the drug sensitivity data. The scalable bi-convex optimization algorithm is used to solve the objective function. Since the $L_{1}$-norm penalty can produce sparsity, the $L_{1}$-norm regularization item is added on the drug-pathway association matrix. By applying the iPaD method on the NCI-60 and Cancer Cell Line Encyclopedia (CCLE) datasets, Li et al. [8] prove that the iPaD method performs better than the iFad method in computational efficiency and identifying drug-pathway association pairs. Since the $L_{2,1}$-norm regularization can penalize each row of the matrix as a whole and can enhance the sparsity among the rows [11], based on this theory, a method named $L_{2,1}$-iPaD ($L_{2,1}$-integrative Penalized Matrix Decomposition) is proposed to identify paired drug-pathway associations [12]. The $L_{1}$-norm penalization can produce scattered and unstructured sparsity matrix, yet $L_{2,1}$-norm penalization can produce structured row sparsity matrix [11]. Moreover, the sum of the $L_{1}$-norm and $L_{2,1}$-norm penalization can produce row structure with intra-row sparsity. In this paper, for the purpose of enhancing the sparsity of the drug-pathway association matrix and improving the performance of the method, we use the sum of the $L_{1}$-norm regularization and $L_{2,1}$-norm regularization instead of the $L_{1}$-norm regularization. In this article, a novel method named “$L_{1}L_{2,1}$-iPaD” is proposed to identify paired drug-pathway associations. Our method has the following advantages. Firstly, for the first time, we propose the $L_{1}L_{2,1}$-iPaD method by replacing the $L_{1}$-norm regularization with the sum of the $L_{1}$-norm regularization and $L_{2,1}$-norm regularization. Secondly, the $L_{1}L_{2,1}$-iPaD method can be used to analyze gene expression and drug sensitivity data. Thirdly, it gives an effective method to identify drug-pathway association pairs. Experimental results in two real datasets prove that the $L_{1}L_{2,1}$-iPaD method is effective.

The remainder of this paper is organized as follows. Firstly, we will introduce two real datasets, give out the experimental results and show the comparison of our method with the state-of-art methods. Then we will provide discussion of this article and outline the future works. And thirdly, we will introduce the notations and definitions in this paper. Finally, we will describe the related works and methodology of the $L_{1}L_{2,1}$-iPaD.

## THE INTRODUCTION FOR DATASETS

### CCLE data set

The CCLE data set is downloaded from the CCLE project, which is a competitive resource to implement a detailed genetic characterization for a large panel of human cancer cell lines. The CCLE project (http://software.broadinstitute.org/software/cprg/?q=node/11) can provide public access analysis, visualization of DNA copy number, mutation data, mRNA expression, and so on, for about 1046 cancer cell lines. The CCLE data set consists of 480 cell lines with drug sensitivity data for 22 drugs and transcription data for 1802 genes covering 58 KEGG pathways. In fact, the CCLE data set in the CCLE project contains 18988 genes and 24 chemical drugs. The detailed data processing can be found in [8]. The drug sensitivities are measured by area over the dose-response curve (‘activity area’). ${EC}_{50}$, $IC_{50}$ and maximum activity area (‘$A_{\max}$’) can also measure drug sensitivities. But activity area has two advantages. Firstly, it has less missing values. Secondly, it can both reflect drug potency and efficacy. But ${EC}_{50}$ and $IC_{50}$ can only reflect drug potency, and $A_{\max}$ can only reflect its efficacy. In this paper, the density of the gene-pathway association prior knowledge matrix $H^{\left(1\right)}$ is 3.95%. And for the drug-pathway association prior knowledge matrix $H^{\left(2\right)}$, we set it to zero.

### NCI-60 data set

The NCI-60 data set contains 57 cell lines with gene expression data for 1863 genes covering 58 KEGG [13] pathways and drug sensitivity data for 101 drugs. The NCI-60 data set is from the NCI-60 project, which provides various type of ‘Omics’ features of 60 cell lines with 9 different cancer types. The gene expression data and the drug sensitivity profiles are downloaded from the CellMiner database [9]. The detailed data processing can be found in [5]. The drug sensitivity data are obtained by $GI_{50}$ values, those values can reflect the potency of drugs. The $GI_{50}$ is defined as the needed concentration for 50% of maximum cell growth inhibition. The drug sensitivity data values are equal to the $\log10\left(GI_{50}\right)$. Higher values mean higher drug sensitivity of the cell lines. Besides, the density of the gene-pathway association prior knowledge matrix $H^{\left(1\right)}$ is 3.95%. And the density of the drug-pathway association prior knowledge matrix $H^{\left(2\right)}$ is 0.51%.

## RESULTS

In this Section, we evaluate the performance of our proposed $L_{1}L_{2,1}$-iPaD method by applying to the CCLE and NCI-60 datasets. To show the effectiveness of our proposed method, we also compare our method with the iPaD and $L_{2,1}$-iPaD methods in this Section.

### The results on the CCLE data set

In this experiment, we first use the five-fold cross-validation to obtain the optimal $\lambda_{1}$ and $\lambda_{2}$ values, and then obtain the sparse drug-pathway association matrix $B^{\left(2\right)}$ corresponding to the optimal parameter values. Similar to the iPaD and $L_{2,1}$-iPaD methods, our method also can assess the relative importance of the coefficients in the drug-pathway association matrix $B^{\left(2\right)}$ via solving the $L_{1}L_{2,1}$-problem for a decreasing λ sequence. For each λ value, we record the order of the coefficients in which they become nonzero. In general, the more important coefficients ought to become nonzero earlier than the less important coefficients. However, this procedure cannot be used to assess the significance of the coefficients. Therefore, we perform permutation test to assess the significance of the coefficients in the drug-pathway association matrix $B^{\left(2\right)}$. In this paper, we run 2000 permutations to obtain the P-values. The P-values of our method, the $L_{2,1}$-iPaD and iPaD methods on the CCLE data set are listed in Table 1, in which the superior results are shown in bold type. In this paper, the known drug-pathway associations are served as the validated information. In Table 1, the drug named Nutlin-3 is related with the Chronic myeloid leukemia pathway. A published paper suggests that Nutlin-3 can up-regulate the expression of Notch1 in both lymphoid and myeloid leukemic cells for a part of the negative feedback antiapoptotic mechanism [14]. Besides, the authors in [15] confirm that IAP inhibition using a small synthetic inhibitor (LBW-242) increases the sensitivity of CML cells to TKI. This drug-pathway association pair is not validated in the CancerResource, but our method can find their associations. And in Table 1, only 5 drug-pathway association pairs are validated in the CancerResource. For the rest 10 drug-pathway associations which are not validated in the CancerResource, we retrieve published papers to prove their associations. Only one association pair is not found from published papers. Besides, similarly, the authors in [16] suggest that cotreatment with LBH589 and 17-AAGcan induce more apoptosis of IM-resistant primary CML-BC and acute myeloidleukemia cells than treatments with either agent alone. And in this paper, our new method also can find that 17-AAG is associated with the Chronic myeloid leukemia pathway. Moreover, the drug-pathway pairs corresponding to nonzero elements in the matrix $B^{\left(2\right)}$ are selected as the identified drug-pathway association pairs. In this experiment, our method identifies 413 drug-pathway pairs that have p-value no more than 0.05, and 70 drug-pathway pairs are validated in the CancerResource database. The $L_{2,1}$-iPaD method identifies 368 drug-pathway pairs that have p-value no more than 0.05, and 66 drug-pathway pairs are validated in the CancerResource database. However, iPaD identifies 88 drug-pathway pairs that have p-value no more than 0.05, and only 25 drug-pathway pairs are validated in the CancerResource database. When we set the P-value cutoff as 0.005, 51 drug-pathway association pairs are identified by the iPaD method, with 16 association pairs validated in the CancerResource database. But for our method and the $L_{2,1}$-iPaD method, 53 drug-pathway association pairs are identified, with 16 association pairs validated in the CancerResource database. In addition, in [8], we can easily find that the results of iFad and GSEA methods are poorer than the iPaD method, so in this paper, we does not compare our method with the iFad and GSEA methods.

**Table 1:** The top 15 identified drug-pathway associations on CCLE data set by $L_{1}L_{2,1}$-iPaD, $L_{2,1}$-iPaD and iPaD methods

Drug	Pathway	$L_{1}L_{2,1}$-iPaD	$L_{2,1}$-iPaD	-iPaD	Validated
		P-value			
Nutlin-3	Chronic myeloid leukemia	**0**	1.74E-43	1.09E-17	CR
PD-0332991	Chronic myeloid leukemia	**0**	6.93E-41	3.16E-13	CR
LBW242	Chronic myeloid leukemia	**1.51E-45**	2.80E-44	8.08E-17	[28]
17-AAG	Chronic myeloid leukemia	**5.52E-45**	9.46E-43	3.41E-16	[16]
L-685458	Chronic myeloid leukemia	**6.81E-44**	4.33E-43	3.32E-19	[29]
AZD0530	Colorectal cancer	**1.46E-43**	1.62E-41	8.81E-13	[30]
PHA-665752	Chronic myeloid leukemia	**1.03E-41**	1.09E-40	3.41E-16	[31]
Paclitaxel	Chronic myeloid leukemia	**2.11E-40**	2.14E-38	4.58E-12	[32]
AZD0530	Chronic myeloid leukemia	**5.86E-40**	7.12E-38	4.76E-18	CR
PD-0325901	Thyroid cancer	**1.25E-28**	3.59E-12	2.57E-05	[33]
ZD-6474	Chronic myeloid leukemia	**1.52E-22**	1.62E-21	2.36E-10	[34]
RAF265	ECM-receptor interaction	**2.19E-17**	1.26E-15	2.32E-04	Unfound
AZD0530	ErbB signaling pathway	**1.13E-16**	4.41E-16	5.10E-06	CR
Erlotinib	Chronic myeloid leukemia	**3.71E-15**	5.69E-15	2.34E-08	[35]
Nilotinib	ErbB signaling pathway	**4.00E-14**	1.23E-13	1.80E-05	CR

Then we compute the identification and verification rates of drug-pathway association pairs on the CCLE data set. Specifically, we make the ratio of number of identification (or verification) and total number of drug-pathway pairs as the identification (or verification) rate. The identification and verification rates of drug-pathway association pairs on the CCLE data set for the $L_{1}L_{2,1}$-iPaD, $L_{2,1}$-iPaD and iPaD methods can be found in Table 2 and Table 3. It is obvious that our method can identify more drug-pathway association pairs than other existing methods.

**Table 2:** The identification and verification rates on CCLE data set with P-value<0.005

Method	Number of identification	Number of verification	Verification rate	Identification rate
$L_{1}L_{2,1}$-iPaD	**53**	**16**	**0.0125**	**0.0415**
$L_{2,1}$-iPaD	**53**	**16**	**0.0125**	**0.0415**
iPaD	51	**16**	**0.0125**	0.0399

**Table 3:** The identification and verification rates on CCLE data set with P-value<0.05

Method	Number of identification	Number of verification	Verification rate	Identification rate
$L_{1}L_{2,1}$-iPaD	**413**	**70**	**0.0549**	**0.3237**
$L_{2,1}$-iPaD	368	66	0.0517	0.2884
iPaD	88	25	0.0196	0.0689

### The results on the NCI-60 data set

Similar to the $L_{2,1}$-iPaD and iPaD methods, we also apply our method to the NCI-60 data set. For the NCI-60 data set, we also run 2000 permutation to evaluate the significance of the coefficients in the drug-pathway association matrix $B^{\left(2\right)}$. The P-values of our method, the $L_{2,1}$-iPaD and iPaD methods on the NCI-60 data set are listed in Table 4. The authors in [17] prove that the mechanism of action of the EA derivatives prepared in this study is more complex than the inhibition of glutathione S-transferase p ascribed as unique effect to EA and might help to overcome tumor resistances. And in Table 4, Melphalan is associated with T cell receptor signaling pathway. A study published in 2003 suggests that Melphalan can control the expression of T cell receptor signaling pathway [18]. In Table 4, only 6 drug-pathway association pairs are validated in the CancerResource. For the rest 9 drug-pathway associations which are not validated in the CancerResource, we retrieve published papers to prove their associations. Only one pair associations are not found from published papers. So, our method can also identify associations, which are not validated in CancerResource, in the NCI-60 data set between drugs and pathways. For example, a paper studied in 2000 writes that an *in vitro* study the effects of MPA (Mycophenolic acid) on human peripheral blood lymphocyte activation markers and on cell cycle characteristics are investigated [19]. Moreover, the drug-pathway pairs corresponding to nonzero elements in the matrix $B^{\left(2\right)}$ are selected as the identified drug-pathway association pairs. In this experiment, our method identifies 593 drug-pathway pairs that have p-value no more than 0.05, and 163 drug-pathway pairs are validated in the CancerResource database. The L2,1-iPaD method identifies 562 drug-pathway pairs that have p-value no more than 0.05, and 163 drug-pathway pairs are validated in the CancerResource database. However, iPaD identifies 247 drug-pathway pairs that have p-value no more than 0.05, and only 74 drug-pathway pairs are validated in the CancerResource database. And when we set the P-value cutoff as 0.005, the results of our method is similar to the L2,1-iPaD method, but the number of identification and verification are higher than the iPaD method. Similar to the CCLE data set, we also compute the identification and verification rates of drug-pathway association pairs on the NCI-60 data set. The identification and verification rates of drug-pathway association pairs on the NCI-60 data set for the $L_{1}L_{2,1}$-iPaD, $L_{2,1}$-iPaD and iPaD methods can be found in Table 5 and Table 6.

**Table 4:** The top 15 identified drug-pathway associations on NCI-60 data set by $L_{1}L_{2,1}$-iPaD, $L_{2,1}$-iPaD and iPaD methods

Drug	Pathway	$L_{1}L_{2,1}$-iPaD	$L_{2,1}$-iPaD	iPaD	Validated
		P-value			
Hydroxyurea	Neuroactive ligand-receptor interation	**0**	**0**	NAN	[36]
Rebeccamycin	T cell receptor signaling pathway	**1.78E-17**	4.12E-16	4.65E-10	Unfound
Tiazofurin	Cell cycle	**7.70E-12**	8.19E-11	7.54E-07	CR
Selenazofurin	Cell cycle	**8.27E-11**	1.75E-10	2.78E-07	CR
Mycophenolic acid	Cell cycle	**9.02E-11**	2.61E-10	2.52E-06	[19]
Lucanthone	Tight junction	2.06E-08	**9.97E-09**	4.31E-06	CR
Primaquine	Neuroactive ligand-receptor interation	**4.46E-08**	1.14E-06	2.69E-04	[37]
Ethacrynic acid	Glutathione metabolism	**1.17E-07**	2.29E-02	6.36E-03	CR
Aminoglutethimide	Primary immunodeficiency	**6.55E-07**	1.30E-06	1.16E-04	[38]
Diallyl disulfide	Acute myeloid leukemia	**8.76E-07**	8.13E-06	8.41E-05	[39]
Bleomycin	Focal adhesion	**1.46E-06**	1.17E-05	4.56E-04	[40]
Geldanamycin	Gap junction	**1.46E-06**	7.89E-06	1.87E-04	[41]
Melphalan	T cell receptor signaling pathway	**3.76E-06**	2.64E-05	6.16E-04	CR
Lomustine	Tight junction	**6.74E-06**	1.06E-05	2.64E-04	CR
Vitamin K3	Metabolism of xenobiotics by cytochrome P450	**9.98E-06**	2.22E-05	2.71E-04	[42]

**Table 5:** The identification and verification rates on NCI-60 data set with P-value<0.05

Method	Number of identification	Number of verification	Verification rate	Identification rate
$L_{1}L_{2,1}$-iPaD	**593**	**163**	**0.0278**	**0.1012**
$L_{2,1}$-iPaD	562	**163**	**0.0278**	0.0959
iPaD	247	74	0.0126	0.0422

**Table 6:** The identification and verification rates on NCI-60 data set with P-value<0.005

Method	Number of identification	Number of verification	Verification rate	Identification rate
$L_{1}L_{2,1}$-iPaD	**89**	**34**	**0.0058**	**0.0152**
$L_{2,1}$-iPaD	**89**	33	0.0056	**0.0152**
iPaD	72	26	0.0044	0.0122

## DISCUSSION

Identifying drug-targets is a momentous issue for the bioinformatics and a crucial step for the drug discovery. In this paper, we proposed a novel method named “$L_{1}L_{2,1}$-iPaD” to identify the paired drug-pathway associations, and used it to jointly analyze the gene expression and drug sensitivity data. In addition, for the $L_{1}L_{2,1}$-iPaD method, two parameters need to adjust. So, we use five-fold cross-validation to choose the optimal parameters. Besides, we perform permutation test to assess the significance of the identified drug-pathway association pairs. In order to evaluate the performance of our method, we apply it to two real datasets, including the CCLE and NCI-60 datasets. Moreover, we compare our method with the iPaD and $L_{2,1}$-iPaD methods. The experimental results demonstrate that our method is superior to the iPaD and $L_{2,1}$-iPaD methods. Our method can identify more drug-pathway association pairs which are validated in the CancerResource database than other methods. Besides, for the rest drug-pathway associations which are not validated in the CancerResource database, we retrieve published papers to prove their associations.

With the development of the high-throughput technology, more and more genomic data sets are generated from various fields. At present, one of our central task is to develop the feasible and efficient method to analyze them. Our method is one of the useful ways for identifying drug-pathway association pairs. In the future, we will develop more efficient and robust methods to jointly analyze high dimensional data and to solve the computational challenges.

## NOTATIONS AND DEFINITIONS

In this Section, we summarize the definition of norms, which will be used in the methods. Given a matrix $M=\left(m_{i,j}\right)$, $m^{i}$ denotes the i-th row of the matrix Μ.

For the matrix $Μ\in R^{d\times n}$, the $L_{1}$-norm of a matrix was first presented in [20], whose definition can be written as

$${\left\|M\right\|}_{1}=\sum_{i=1}^{d}\sum_{j=1}^{n}\left|m_{i,j}\right|.$$ (1)

The Frobenius norm of the matrix Μ can be defined as

$${\left\|M\right\|}_{F}=\sqrt{\sum_{i=1}^{d}\sum_{j=1}^{n}m_{i,j}^{2}}=\sqrt{\sum_{i=1}^{d}{\left\|m^{i}\right\|}_{2}^{2}}.$$ (2)

And the $L_{2,1}$-norm of a matrix was first introduced in [21]. Until now, the $L_{2,1}$-norm has been used in many fields, such as the feature extraction [22] and the image processing [23, 24] etc. The $L_{2,1}$-norm of the matrix Μ is defined as

$${\left\|M\right\|}_{2,1}=\sum_{i=1}^{d}\sqrt{\sum_{j=1}^{n}m_{i,j}^{2}}=\sum_{i=1}^{d}{\left\|m^{i}\right\|}_{2}.$$ (3)

The $L_{r,p}$-norm of the matrix Μ can be written as follows [16]:

$${\left\|M\right\|}_{r,p}={\left(\sum_{i=1}^{d}{\left(\sum_{j=1}^{n}\left|m_{i,j}\right|\right)}^{p/r}\right)}^{1/p}={\left({\sum_{i=1}^{d}\left\|m^{i}\right\|}_{r}^{p}\right)}^{1/p}.$$ (4)

## RELATED WORKS

### iPaD method

Let $Y^{\left(1\right)}\in R^{N\times G^{\left(1\right)}}$ and $Y^{\left(2\right)}\in R^{N\times G^{\left(2\right)}}$ denote drug sensitivity data and transcription data matrices, respectively. N is the number of the samples (usually cell lines). $G^{\left(1\right)}$ and $G^{\left(2\right)}$ are the number of genes and number of drugs, respectively. Besides, $X\in R^{N\times K}$ denotes a pathway activity matrix, that is, it indicates the activity level of K pathways in the N samples. For the traditional iPaD method [8], the authors decompose the matrix $Y^{\left(1\right)}$ into the matrices X and $B^{\left(1\right)}$, and the matrix $Y^{\left(2\right)}$ into the matrices X and $B^{\left(2\right)}$. Therefore, the model can be introduced as follows:

$$\begin{array}{l} Y^{\left(1\right)}=XB^{\left(1\right)}+E^{\left(1\right)}\\ Y^{\left(2\right)}=XB^{\left(2\right)}+E^{\left(2\right)}, \end{array}$$ (5)

where $E^{\left(1\right)}$ and $E^{\left(2\right)}$ are the error matrices. $B^{\left(1\right)}$ and $B^{\left(2\right)}$ denote the gene-pathway association and drug-pathway association matrices, respectively. In general, the model (5) can be formulated as follows:

$$\underset{X,B^{\left(1\right)},B^{\left(2\right)}}{\min}{\left\|Y^{\left(1\right)}-XB^{\left(1\right)}\right\|}_{F}^{2}+{\left\|Y^{\left(2\right)}-XB^{\left(2\right)}\right\|}_{F}^{2},$$ (6)

where ${\left\|\cdot \right\|}_{F}$ denotes the Frobenius norm.

For the Eq.(6), the optimization model of iPaD method [8] is defined as follows:

$$\begin{array}{l} \underset{X,B^{\left(1\right)},B^{\left(2\right)}}{min}{\left\|Y^{\left(1\right)}-XB^{\left(1\right)}\right\|}_{F}^{2}+{\left\|Y^{\left(2\right)}-XB^{\left(2\right)}\right\|}_{F}^{2}+\lambda {\left\|B^{\left(2\right)}\right\|}_{1}\\ \text{ subject to }\sum_{i}X_{i,j}^{2}\leq 1,\forall j=1,\ldots ,K,\\ \;B_{i,j}^{\left(1\right)}=0,\forall \left(i,j\right):H_{i,j}^{\left(1\right)}=0, \end{array}$$ (7)

where ${\left\|B^{\left(2\right)}\right\|}_{1}$ is the $L_{1}$-norm of the matrix $B^{\left(2\right)}$. λ is a crucial parameter and used to adjust the sparsity of the matrix $B^{\left(2\right)}$. In general, the bigger the value of λ is, the more sparse the matrix $B^{\left(2\right)}$ is. Since a drug is usually related to a few pathways, the matrix $B^{\left(2\right)}$ may be sparse. Because the $L_{1}$-norm can produce sparsity, the authors in [8] add the $L_{1}$-norm constraint on the matrix $B^{\left(2\right)}$. And, the prior knowledge matrix $H^{\left(1\right)}\in {\left\{0,1\right\}}^{K\times G^{\left(1\right)}}$ is an indicating matrix, that is, the matrix $H^{\left(1\right)}$ is used to indicate elements in the matrix $B^{\left(2\right)}$. If $H_{i,j}^{\left(1\right)}=1$, it indicates that the j-th gene is associated with i-th pathway. However, if $H_{i,j}^{\left(1\right)}=0$, it indicates that the j-th gene is not associated with the i-th pathway. The authors in [8] assume that the known gene-pathway associations are complete, so, they pay attention to discover the novel drug-pathway associations. Therefore, the second constraint condition is used to incorporate known gene-pathway associations. Besides, the first constraint condition is used to guarantee that the model is identical.

### $L_{2,1}$-iPaD method

Since the $L_{2,1}$-norm penalty can produce rows sparsity [25], another effective method named $L_{2,1}$-iPaD is proposed to identify the novel drug-pathway association pairs [12]. In this paper, the $L_{2,1}$-norm regularization is used to replace the $L_{1}$-norm regularization to enforce sparse constraint on rows. And it modifies the optimization problem (7) as follows:

$$\begin{array}{l} \underset{X,B^{\left(1\right)},B^{\left(2\right)}}{min}{\left\|Y^{\left(1\right)}-XB^{\left(1\right)}\right\|}_{F}^{2}+{\left\|Y^{\left(2\right)}-XB^{\left(2\right)}\right\|}_{F}^{2}+\lambda {\left\|B^{\left(2\right)}\right\|}_{2,1}\\ \text{ subject to }\sum_{i}X_{i,j}^{2}\leq 1,\forall j=1,\ldots ,K;\\ \;B_{i,j}^{\left(1\right)}=0,\forall \left(i,j\right):H_{i,j}^{\left(1\right)}=0, \end{array}$$ (8)

where ${\left\|B^{\left(2\right)}\right\|}_{2,1}$ denotes the $L_{2,1}$-norm of the matrix$B^{\left(2\right)}$.

## METHODOLOGY

Since the gene-pathway association information is available and complete, the central interest lies in the inference of the matrix $B^{\left(2\right)}$, that is, the paired drug-pathway associations. We further consider enhancing the sparsity of the matrix $B^{\left(2\right)}$. Thus, we add the $L_{1}$-norm regularization to impose the penalty among all elements in the matrix $B^{\left(2\right)}$ and propose our new $L_{1}L_{2,1}$-iPaD method. The objective function of the $L_{1}L_{2,1}$-iPaD method is defined as follows:

$$\begin{array}{l} \underset{X,B^{\left(1\right)},B^{\left(2\right)}}{min}{\left\|Y^{\left(1\right)}-XB^{\left(1\right)}\right\|}_{F}^{2}+{\left\|Y^{\left(2\right)}-XB^{\left(2\right)}\right\|}_{F}^{2}+\lambda_{1}{\left\|B^{\left(2\right)}\right\|}_{1}+\lambda_{2}{\left\|B^{\left(2\right)}\right\|}_{2,1}\\ \text{ subject to }\sum_{i}X_{i,j}^{2}\leq 1,\forall j=1,\ldots ,K,\\ \;B_{i,j}^{\left(1\right)}=0,\forall \left(i,j\right):H_{i,j}^{\left(1\right)}=0, \end{array}$$ (9)

where $\lambda_{1}$ and $\lambda_{2}$ are two adjustable parameters, which can increase or decrease the sparsity of the matrix $B^{\left(2\right)}$. In general, the bigger the $\lambda_{1}$ and $\lambda_{2}$ are, the more sparse the matrix $B^{\left(2\right)}$ is. Optimization problem (9) is convex. That is, when we fix $B^{\left(1\right)}$ and $B^{\left(2\right)}$, optimizing X is a convex problem, and when we fix X, optimizing $B^{\left(1\right)}$ and $B^{\left(2\right)}$ are both convex optimization subproblems. Since it is difficult to directly obtain the solution, an effective method is proposed to solve the optimization problem in Eq.(9).

### Optimizing X


$$\begin{array}{l} \underset{X}{min}{\left\|Y-XB\right\|}_{F}^{2}\\ \text{subject to }\sum_{i}X_{i,j}^{2}\leq 1,\forall j=1,\ldots ,K, \end{array}$$ (10)

where $Y=\left[Y^{\left(1\right)},Y^{\left(2\right)}\right]$ and $B=\left[B^{\left(1\right)},B^{\left(2\right)}\right]$. The iPaD method [8] used the traditional gradient descent method to optimize X. Here, we also apply the gradient descent method to solve this problem. The objective function of Eq.(10) can be written as follows:

$$\begin{array}{l} {\left\|Y-XB\right\|}_{F}^{2}\\ =\mathrm{Tr}\left[{\left(Y-XB\right)}^{T}\left(Y-XB\right)\right]. \end{array}$$ (11)

By computing the derivative of Eq.(11), we can obtain

$$\begin{array}{c} \frac{\partial {\left\|Y-XB\right\|}_{F}^{2}}{\partial X}=-2\left(Y-XB\right)B^{T}\\ =2\left(XBB^{T}-YB^{T}\right). \end{array}$$ (12)

So that X can be updated by

$$X_{k+1}=X_{k}-2\mu \left(XBB^{T}-YB^{T}\right),\;k=0,1,2,\cdots .$$ (13)

Here, μ is the iteration step size. At every iteration, we check whether $X_{k+1}$ is located in the feasible fields $\left\{X:\sum_{i}X_{i,j}^{2}\leq 1,\forall j=1,\ldots ,K\right\}$. If ${\text{X}}_{k+1}$ satisfies this condition, we perform next step, otherwise, we make ${\text{X}}_{k+1}={\text{X}}_{k+1}/\left\|{\text{X}}_{k+1}\right\|$. The Nesterov's method [26] can achieve a convergence rate becoming $1/t^{2}$ (The original convergence rate is 1/t). So, when we update X, we also apply this method to quicken the convergence speed.

### Optimizing $B^{\left(1\right)}$


$$\begin{array}{l} \underset{B^{\left(1\right)}}{min}{\left\|Y^{\left(1\right)}-XB^{\left(1\right)}\right\|}_{F}^{2}\\ \text{subject to }B_{i,j}^{\left(1\right)}=0,\forall \left(i,j\right):H_{i,j}^{\left(1\right)}=0. \end{array}$$ (14)

Least squares method is a common optimization algorithm to solve the unconstrained optimization problems. In order to use the prior knowledge matrix $H^{\left(1\right)}$ to optimize $B^{\left(1\right)}$, similar to [8], we also decompose the original problem into $G^{\left(1\right)}$ OLS (ordinary least squares) problems. That is, we optimize each column of $B^{\left(1\right)}$, separately. The problem (14) can be introduced as follows:

$$\begin{array}{l} \text{ For }q\in \left\{1,2,\cdots ,G^{\left(1\right)}\right\},\\ \underset{B_{:,q}^{\left(1\right)}}{min}{\left\|Y_{:,q}^{\left(1\right)}-X_{:,H_{:,q}^{\left(1\right)}}B_{H_{:,q}^{\left(1\right)},q}^{\left(1\right)}\right\|}_{F}^{2}, \end{array}$$ (15)

where $Y_{:,q}^{\left(1\right)}$ is a vector with the elements corresponding to the q-th column of the matrix $Y^{\left(1\right)}$, and $X_{:,H_{:,q}^{\left(1\right)}}$ denotes the sub-matrix of the matrix X, which is composed of the columns corresponding to the non-zero parts of the prior knowledge matrix $H_{:,q}^{\left(1\right)}$. And $B_{H_{:,q}^{\left(1\right)},q}^{\left(1\right)}$ is a vector with the q-th column vector of $B^{\left(1\right)}$ corresponding to the non-zero parts of the prior knowledge matrix $H_{:,q}^{\left(1\right)}$.

### Optimizing $B^{\left(2\right)}$

For optimizing $B^{\left(2\right)}$, we observe each column of the matrix $B^{\left(2\right)}$ and decompose the original problem into $G^{\left(2\right)}$ sum of $L_{1}$-norm and $L_{2,1}$-norm minimization problems:

$$\begin{array}{l} \text{ For }q\in \left\{1,2,\cdots ,G^{\left(2\right)}\right\},\\ \underset{B_{:,q}^{\left(2\right)}}{min}{\left\|Y_{:,q}^{\left(2\right)}-XB_{:,q}^{\left(2\right)}\right\|}_{2}^{2}+\lambda_{1}{\left\|B_{:,q}^{\left(2\right)}\right\|}_{1}+\lambda_{2}{\left\|B_{:,q}^{\left(2\right)}\right\|}_{2,1}. \end{array}$$ (16)

In order to use the prior knowledge on the drug-pathway associations (matrix $H^{\left(2\right)}$), we add the $L_{2}$-norm on the matrix $B^{\left(2\right)}$. Thus, the problem (16) can be rewritten as follows:

$$\begin{array}{l} \text{ For }q\in \left\{1,2,\cdots ,G^{\left(2\right)}\right\},\\ \underset{B_{:,q}^{\left(2\right)}}{min}{\left\|Y_{:,q}^{\left(2\right)}-XB_{:,q}^{\left(2\right)}\right\|}_{2}^{2}+\lambda_{1}{\left\|B_{\left(1-H_{:,q}^{\left(2\right)}\right),q}^{\left(2\right)}\right\|}_{1}+\lambda_{2}\left({\left\|B_{\left(1-H_{:,q}^{\left(2\right)}\right),q}^{\left(2\right)}\right\|}_{2,1}+{\left\|B_{H_{:,q}^{\left(2\right)},q}^{\left(2\right)}\right\|}_{2}\right). \end{array}$$ (17)

Similar to the prior information matrix $H^{\left(1\right)}$, $H_{i,j}^{\left(2\right)}\in {\left\{0,1\right\}}^{K\times G^{\left(2\right)}}$ is also a prior knowledge matrix, which can indicate drug-pathway association matrix $B^{\left(2\right)}$. $\lambda_{1}$ and $\lambda_{2}$ are two regulable parameters which are used to control the sparsity of the paired drug-pathway association matrix $B^{\left(2\right)}$.

For the part of $B^{\left(2\right)}$ being indicated by $H^{\left(2\right)}$, the problem is written as follows:

$$\underset{B^{\left(2\right)}}{min}{\left\|Y^{\left(2\right)}-XB^{\left(2\right)}\right\|}_{F}^{2}+\lambda_{2}{\left\|B^{\left(2\right)}\right\|}_{2}^{2},$$ (18)

where we omit the symbol of the objective function. The objective function can be converted into the following equation:

$$\begin{array}{c} J_{1}\left(B^{\left(2\right)}\right)={\left\|Y^{\left(2\right)}-XB^{\left(2\right)}\right\|}_{F}^{2}+\lambda_{2}{\left\|B^{\left(2\right)}\right\|}_{2}^{2}\\ =\mathrm{Tr}\left[\left(Y^{\left(2\right)}-XB^{\left(2\right)}\right){\left(Y^{\left(2\right)}-XB^{\left(2\right)}\right)}^{T}\right]+\lambda_{2}\mathrm{Tr}\left({\left(B^{\left(2\right)}\right)}^{T}IB^{\left(2\right)}\right), \end{array}$$ (19)

where I is a unit matrix with the size of K×K, and $J_{1}\left(\cdot \right)$${\text{J}}_{1}\;\left({\text{B}}^{\left(2\right)}\right)$ is an auxiliary function. Then we compute the derivative of $J_{1}\left(B^{\left(2\right)}\right)$ and set its result to zero,

$$\begin{array}{c} \frac{\partial J_{1}\left(B^{\left(2\right)}\right)}{\partial B^{\left(2\right)}}=-2X^{T}Y^{\left(2\right)}+2\left(X^{T}X+\lambda_{2}I\right)B^{\left(2\right)}\\ =0. \end{array}$$ (20)

Hence, we can obtain:

$$B^{\left(2\right)}={\left(X^{T}X+\lambda_{2}I\right)}^{-1}X^{T}Y^{\left(2\right)}.$$ (21)

For the part of $B^{\left(2\right)}$ being indicated by $1-H^{\left(2\right)}$, we will provide a novel and available method to obtain the interesting drug-pathway association matrix $B^{\left(2\right)}$. The optimization problem can be described as follows:

$$\begin{array}{l} \underset{B^{\left(2\right)}}{min}{\left\|Y^{\left(2\right)}-XB^{\left(2\right)}\right\|}_{F}^{2}+\lambda_{1}{\left\|B^{\left(2\right)}\right\|}_{1}+\lambda_{2}{\left\|B^{\left(2\right)}\right\|}_{2,1}\\ =\underset{B^{\left(2\right)}}{min}{\left\|Y^{\left(2\right)}-XD^{-1/2}D^{1/2}B^{\left(2\right)}\right\|}_{F}^{2}+\lambda_{1}\mathrm{Tr}\left[{\left(B^{\left(2\right)}\right)}^{T}\tilde{D}B^{\left(2\right)}\right]\;+\lambda_{2}\mathrm{Tr}\left[{\left(B^{\left(2\right)}\right)}^{T}D^{1/2}D^{1/2}B^{\left(2\right)}\right], \end{array}$$ (22)

where D is a diagonal matrix with the i-th diagonal element as $d_{ii}={1/2\left\|{\left(B^{\left(2\right)}\right)}^{i}\right\|}_{2}$, and $\tilde{D}$ is also a diagonal matrix with the i-th diagonal element as $\tilde{d_{ii}}=1/2\left|{\left(B^{\left(2\right)}\right)}_{i,j}\right|$.

In order to simplify the optimization problem (22), we set $X_{1}=XD^{-1/2}$ and $B_{1}=D^{1/2}B^{\left(2\right)}$. Then, the problem (22) can be equivalent to the following minimization problem:

$$\underset{B_{1}}{min}{\left\|Y^{\left(2\right)}-X_{1}B_{1}\right\|}_{F}^{2}+\lambda_{1}\mathrm{Tr}\left[{\left(B_{1}\right)}^{T}D^{-1/2}\tilde{D}D^{-1/2}B_{1}\right]+\lambda_{2}\mathrm{Tr}\left[{\left(B_{1}\right)}^{T}B_{1}\right].$$ (23)

The objective function is equal to the following equation:

$$J_{2}\left(B_{1}\right)={\left\|Y^{\left(2\right)}-X_{1}B_{1}\right\|}_{F}^{2}+\lambda_{1}\mathrm{Tr}\left[{\left(B_{1}\right)}^{T}D^{-1/2}\tilde{D}D^{-1/2}B_{1}\right]\;+\lambda_{2}\mathrm{Tr}\left[{\left(B_{1}\right)}^{T}B_{1}\right].$$ (24)

And then we compute the derivative of $J_{2}\left(B_{1}\right)$, then set its result to zero, we have:

$$\begin{array}{c} \frac{\partial J_{2}\left(B_{1}\right)}{\partial B_{1}}=2\left[{\left(X_{1}\right)}^{T}X_{1}+\lambda_{2}I+\lambda_{1}D^{-1/2}\tilde{D}D^{-1/2}\right]B_{1}-2{\left(X_{1}\right)}^{T}Y^{\left(2\right)}\\ =0. \end{array}$$ (25)

So, we can obtain:

$$B_{1}={\left[\left(X_{1}{}^{T}X_{1}+\lambda_{2}I+\lambda_{1}D^{-1/2}\tilde{D}D^{-1/2}\right)\right]}^{-1}X_{1}{}^{T}Y^{\left(2\right)}.$$ (26)

Therefore, we finally compute $B^{\left(2\right)}$ by optimizing $B_{1}$, that is, $B^{\left(2\right)}=D^{-1/2}B_{1}$. We sum up the $L_{1}L_{2,1}$-iPaD method as the Algorithm 1. Besides, in this paper, we also use the soft-impute algorithm to deal with the missing values problems in solving X. The detailed steps can be found from the iPaD [8] and $L_{2,1}$-iPaD methods [12].

**Algorithm 1:** The alternating updating algorithm for the $L_{1}L_{2,1}$-iPaD method

Data Input: $Y^{\left(1\right)},\;Y^{\left(2\right)},\;H^{\left(1\right)}$ Parameter: $\lambda_{1},\;\lambda_{2}$ Output: $B^{\left(2\right)}$
Initialization: set $B^{\left(1\right)}=H^{\left(1\right)}$ and set $B^{\left(2\right)}=0$. Optimization: (1). Optimize X: $\begin{array}{l} X=\arg\underset{X}{min}{\left\|Y-XB\right\|}_{F}^{2}\\ s.t.\sum_{i}X_{i,j}^{2}\leq 1,\forall j=1,\ldots ,K, \end{array}$ where $Y=\left[Y^{\left(1\right)},Y^{\left(2\right)}\right]$ and $B=\left[B^{\left(1\right)},B^{\left(2\right)}\right]$. (2). Optimize $B^{\left(1\right)}$: $\begin{array}{l} B^{\left(1\right)}=\arg\underset{B^{\left(1\right)}}{min}{\left\|Y^{\left(1\right)}-XB^{\left(1\right)}\right\|}_{F}^{2}\\ s.t.\;B_{i,j}^{\left(1\right)}=0,\forall \left(i,j\right):H_{i,j}^{\left(1\right)}=0. \end{array}$ (3). Optimize $B^{\left(2\right)}$: $B^{\left(2\right)}=\underset{B^{\left(2\right)}}{argmin}{\left\|Y^{\left(2\right)}-XB^{\left(2\right)}\right\|}_{F}^{2}+\lambda_{1}{\left\|B^{\left(2\right)}\right\|}_{1}+\lambda_{2}{\left\|B^{\left(2\right)}\right\|}_{2,1}.$ (4). Repeat step (1), (2) and (3) until convergence.

### Parameter selection and significance test

Compared with the iPaD and $L_{2,1}$-iPaD methods, our new $L_{1}L_{2,1}$-iPaD method has two adjustable parameters ($\lambda_{1}$ and $\lambda_{2}$), which can control the sparsity of the drug-pathway association matrix $B^{\left(2\right)}$. In this paper, We perform five-fold cross-validation to find the suitable parameters. In the five-fold cross-validation, we divide the matrix $Y^{\left(2\right)}$ into 5 folds. At each round of the cross-validation, we make each of the 5 folds as missing values, and the rest of 4 folds as training data. It is obvious that it is difficult to find the optimal parameters $\lambda_{1}$ and $\lambda_{2}$, simultaneously. Therefore, we solve the $L_{1}L_{2,1}$-iPaD problem to produce a $\lambda_{1}$ sequence by fixing $\lambda_{2}$, and then we use five-fold cross-validation to find an optimal parameter $\lambda_{1}$. We treat the minimum residual sum of squares (RSS) corresponding to the $\lambda_{1}$ value as the optimal value. Similarly, we find an optimal parameter $\lambda_{2}$ by fixing $\lambda_{1}$. Since our method is a sparse optimization algorithm, there are many zero coefficients in the matrix $B^{\left(2\right)}$. We usually treat those nonzero coefficients as the identified core drug-pathway association pairs. After searching for the optimal parameters, we perform permutation test [27] to assess the significance of the coefficients. We first obtain the estimated value of the matrix $B^{\left(2\right)}$, and then compute the P-value of the coefficient for the matrix $B^{\left(2\right)}$, the computational formula is as follows:

$$P_{i,j}=\frac{1}{T}\sum_{t=1}^{T}\left(\left|{\tilde{B}}_{i,j}^{\left(2\right)\left(t\right)}\right|\geq \left|{\overset{\wedge }{B}}_{i,j}^{\left(2\right)}\right|\right),$$ (27)

where ${\tilde{B}}_{i,j}^{\left(2\right)\left(t\right)}$ denotes the t-th permutation estimated values of the matrix $B^{\left(2\right)}$, and T is the total number of permutations, ${\overset{\wedge }{B}}_{i,j}^{\left(2\right)}$ is the estimated values of the matrix $B^{\left(2\right)}$ in the original data.
